# Serum calcitonin estimation in medullary thyroid cancer: basal or stimulated levels?

**DOI:** 10.1186/1756-6614-6-S1-S4

**Published:** 2013-03-14

**Authors:** Chantal Daumerie, Dominique Maiter, Damien Gruson

**Affiliations:** 1 Department of Endocrinology, Université Catholique de Louvain, Cliniques Universitaires Saint-Luc, Avenue Hippocrate 10, 1200 Brussels, Belgium; 2 Department of Biochemistry, Université Catholique de Louvain, Cliniques Universitaires Saint-Luc, Avenue Hippocrate 10, 1200 Brussels, Belgium

## Abstract

Calcitonin (Ct) is a tumour marker essential for the diagnosis and follow-up of medullary thyroid cancer (MTC). Accurate and consistent measurements of serum Ct are of critical importance. Ct measurements by different methods can differ, leading to difficulties in the interpretation of results. Second generation assays for Ct have been developed and are now available in clinical laboratories. However, the lack of standardization for Ct assays remains a common problem with Ct assays. The reference interval and reliability should be carefully defined.

The role of stimulated Ct for the diagnosis and follow-up of MTC should also be pointed out as the pentagastrin test is no more available in all countries. However, the stimulated test remains very useful to exclude MTC if the basal Ct serum level is in the grey zone (15-20 ng/L), after surgery to confirm the complete cure. A residual response after surgery could indicate a need for aggressive surgery or - in case of metastatic disease - could suggest the prognosis.

High-dose Ca test (2.5mg/kg) seems to be a reliable and effective test for the diagnosis and follow-up of MTC. It seems more potent than pentagastrin with fewer side effects. The threshold able to discriminate healthy subjects from C-cell hyperplasia (CCH) cases for the stimulated Ct concentration is 184 ng/L for women and 1620 ng/L for men.

As stimulated Ca test will eventually replace the pentagastrin test, there is a need to confirm or to modify the threshold identified for each assay individually.

## Introduction

Medullary thyroid carcinoma (MTC) originates from thyroid C cells, which secrete calcitonin (Ct).

Routine measurement of Ct in patients with nodular goitre allows for the preoperative diagnosis of unsuspected MTC, often at a very precocious stadium.

Ct is a 32 amino- acid- polypeptide, in which the disulphide bridges are essential for biological activity. The physiological role of Ct is unknown. The C cells use the same calcium receptor as do parathyroid cells [1], and high calcaemia is their physiological stimulant. Non physiological stimulants include glucagon, β-adrenergic agonists, alcohol, and gastrin [2].

### Basal calcitonin

Accurate and consistent measurements of serum Ct are of critical importance. Ct values measured by different methods can differ, leading to difficulties in interpretation of results

Since 1988, RIA with polyclonal antibodies that recognised both mature Ct monomer and other circulating forms lacking specificity and sensibility, have been used. Second generation automated immunoassays with more specific antibodies are now available for clinical laboratories.

Minimal and mild elevations in serum Ct may be seen in C-Cells hyperplasia, renal failure, autoimmune thyroiditis, and hypercalcaemia. Elevated Ct levels may result from non thyroid neuroendocrine tumours. Falsely low Ct levels may occur in the setting of heterophilic antibodies and from a “hook effects”. Optimally, an individual should be followed using the same Ct assay over time and the laboratory should report which Ct assay is being used. Ct values should also be interpreted in the setting of gender specific reference intervals. Moreover, caution has to be taken in interpreting values in children younger than 3 years.

The effort of standardization for some thyroid related assays is on-going; nevertheless Ct assays remain yet not standardized [3]. Table 1 summarizes the Ct assay performances and data provided by the manufactures. Therefore, the reference interval for a healthy population as well as the reliability should be clearly defined for every Ct assay. The American Thyroid association guidelines do not specify any reference range for Ct but several recent reports have proposed some cut-off points for Ct testing.

**Table 1:** Calcitonin assays performances

Assay	Format	Sample volume	Pretreatment	Within-run CV	Between-run CV	Measuring range	LLD
**Cis-bio**	RIA	200µl	30 min 56°C	6.7% at 10.9 ng/L	5.2% at 21.1 ng/L	1.5 –1530 ng/L	1.5 ng/L
**Diasorin**	Automated chemi-luminescent	150µl	NO	2. 5% at 25 ng/L 3.1% at 160 ng/L	5% at 25 ng/L 4.7% at 160 ng/L	1.0 – 2000 ng/L	4 ng/L
**Siemens**	Automated chemi-luminescent	200 µl	NO	3.4% at 29 ng/L 2.3% at 200 ng/L	4.2% at 29 ng/L 5.5% at 260 g/L	2.0 – 2000 ng/L	2 ng/L
**IBL**	ELISA	100 µl	NO	2.8% at 37 ng/L 2.4% at 260 ng/L	8.6% at 41 ng/L 6.3% at 167 ng/L	1.3 – 790 ng/L	1.3 ng/L
**DSL**	IRMA	150 µL	NO	2.4% at 27 ng/L 5.1% at 390 ng/L	9.0% at 24.3 ng/L 7.8% at 391 ng/L	5 – 500 ng/L	5 ng/L

Doyle reported [4] that the basal Ct (measured by a two-site automated chemiluminescent immunometric assay) was 5 ng/L for men and 5.7 ng/L for women (95^th^ percentile). Rink [5] found that the basal Ct level was 32.9 ng/L for men and 14.6 ng/L for women.

An upper limit of 15 ng/L should be able to rule out MTC and reduce false positive cases [5]. In the range between 15-50 ng/L, the predictive value for detection of MTC was 4%. d’Herbomez et al. [6] suggested a 20 ng/L threshold to limit false positive results and a second control. Colombo et al. [7] demonstrated that the best levels of basal Ct (assayed by chemiluminescence, Immulite 2000) to separate healthy subjects and CCH cases from MTC patients were above 18.7 ng/L in females and above 68 ng/L in males.

However, the question arises whether the decision-making can be reliably based on a single basal Ct measurement for diagnosis in patients with a genetic susceptibility to develop MTC (i.e., activated *RET* oncogene-carriers) or for the follow-up after surgery for a known MTC. This strategy was recently recommended by the American thyroid association as the pentagastrin is now unavailable in many countries [8]. However, even if the ultrasensitive Ct assay will reduce the false negative rate of basal Ct measurements when diagnosing familial MTC and in post-operative follow-up, compared to previously used assays, it has recently been shown that its sensitivity to detect C-cell disease remains lower than a stimulation test [9].

### Indications of calcitonin stimulation tests

The role of stimulated Ct for the diagnosis and follow-up of MTC has recently been pointed out since the classical pentagastrin test is no more available in some European countries.

The stimulation test remained very useful to exclude an MTC in an unaffected individual when basal Ct was in the grey zone (15-50 ng/L) as observed in autoimmune thyroiditis with CCH or in neuroendocrine tumours. Another indication for the stimulation test was to detect residual disease or recurrence after surgery for MTC in patients with low basal Ct levels. Patients who have non-detectable and non-stimulable post-operative Ct at two consecutive follow-up visits are considered disease-free, although they still require yearly follow-up assessments as late recurrence of disease can occur, and there might thus be a need for future complementary surgery. However, it must be remembered that the volume of residual disease is usually very low when only the stimulated Ct level is detectable, and unlikely to be found by imaging until basal Ct is over 150 ng/L [10]. In case of metastatic disease, the response and the peak value could also be indicative of the prognosis. Finally, in genetically predisposed patients with intermediate or low-risk *RET* proto-oncogene mutations, a prophylactic thyroidectomy is usually advised if basal Ct is lower than 10 ng/L and peak Ct (following pentagastrin stimulation test) is between 50 and 100 ng/L.

### Pentagastrin stimulation test

The pentagastrin stimulation test uses a slow intravenous injection of pentagastrin (0.5 µg/kg body weight) over three minutes. Blood samples are obtained at baseline, and two and five minutes after pentagastrin injection.

The cut-off of the pentagastrin-stimutated Ct is still not clear in the literature and depends on the assay used. Verga et al. [11] showed that a peak above 50ng/L indicated a risk of CCH and MTC, while Scheuba et al. [12] found that the probability of having an MTC was 100% if the peak value was higher than 560 ng/L; finally, Elisei et al. [13] determined the lowest peak of Ct for MTC to be 118 ng/L. In chronic renal disease, the peak may reach 400 ng/L. In 2002, the National Academy of Clinical Biochemistry (NACB) has documented that 80% of healthy subjects have a Ct peak lower than 10 ng/L, 15% have a peak between 10 and 30 ng/L and 5% may have a peak between 30 and 50 ng/L [14]. When the peak stimulated Ct was between 50 and 100 ng/L, the risk of diagnosis of C-cell pathology is intermediate and a peak higher than 100 ng/L likely indicates CCH or MTC.

In a recent study, a peak Ct of 275 ng/L determined by IRMA after pentagastrin was able to clearly distinguish patients with MTC from patients with CCH with 100% sensitivity and 89% specificity [15].

It therefore appeared that if stimulated Ct (sCt) values were higher than 200 ng/L, MTC was likely and thyroidectomy and lymphadenectomy required. If sCt reached values between 100 and 200 ng/L, the risk was uncertain. Such values could be indicative of C cell hyperplasia or microscopic MTC. Some would advise surgery, others preferred observation.

### The calcium stimulation test

Due to the unavailability of pentagastrin in many countries, there is a growing interest in the calcium stimulation test.

This test uses an infusion of calcium gluconate (2.5 mg elemental calcium/kg body weight) over 30 seconds administered in fasting state. Blood samples for Ct are obtained at baseline and two and five minutes after the stimulus. High-dose calcium is more effective and a better-tolerated Ct stimulator than pentagastrin.

There are very few data using the calcium stimulation as a confirmatory test in patients with C-cell disease. Cut-off points for the discrimination of healthy subjects, C cell hyperplasia and MTC cases have not been standardized yet. In one study [4] the levels of Ct stimulated after either pentagastrin or calcium were significantly correlated. In this study, calcium stimulated Ct levels above 32.6 ng/L (females) and 192 ng/L (males) had the best accuracy to differentiate normal subjects from patients with C cell hyperplasia and MTC and values above 184 ng/mL in females and above 1620 ng/L in males had the highest accuracy to distinguish healthy subjects and CCH cases from the patients with MTC.

The criteria for abnormal Ct values may vary according to Ct assays and reference values must be defined for every assay. Provided these references ranges are clearly assessed, the indications and usefulness of the high-dose calcium stimulation test should be very similar to the now “old “ pentagastrin test.

## Conclusions

Evaluation of both basal and stimulated Ct may be useful in the diagnosis and follow-up of MTC. Even though ultrasensitive Ct assays have greatly reduced the false negative rate of a basal Ct measurement when diagnosing C-cell disease, its sensitivity remains lower than a stimulation test. The calcium stimulation test may be used in this setting, in particular when basal Ct is in the grey zone or to detect residual disease or early recurrence after surgery for MTC in patients with low basal Ct levels. However, each Ct assay must be evaluated and each laboratory has to define its own reference ranges for basal and stimulated values. Further studies are clearly needed to optimize the Ct thresholds to be used to distinguish patients with C-cell pathology from normal, and to better define the patterns of response in particular conditions, such as in thyroid autoimmune disease and in renal insufficiency.

## List of abbreviations used

Ct: Calcitonin; CCH: C-cell hyperplasia; MTC: medullary thyroid carcinoma; IRMA: immuno radiometric assay; RIA: radio immuno assay.

## Competing interests

No competing interests exist for me and my co-authors.

## Declarations

This article has been published as part of *Thyroid Research* Volume 6 Supplement 1, 2013: European comments on Medullary Thyroid Cancer Management Guidelines of the American Thyroid Association. The full contents of the supplement are available online at http://www.thyroidresearchjournal.com/supplements/6/S1. Publication of this supplement has been supported by the European Thyroid Association-Cancer Research Network.
